# The enhanced cytotoxicity on breast cancer cells by Tanshinone I-induced photodynamic effect

**DOI:** 10.1038/s41598-023-43456-5

**Published:** 2023-10-23

**Authors:** Chen Fengchao, Zhang Siya, Yan Tongtong, Wang Hongquan, Li Jie, Wang Qiang, Subhan Danish, Li Kun

**Affiliations:** 1 Medical Cosmetic Center, Beijing Friendship Hospital, Capital Medical University, Beijing, 100050 People’s Republic of China; 2 Institute of Biomedical and Health Science, School of Life and Health Science, Anhui Science and Technology University, Fengyang, 233100 Anhui People’s Republic of China; 3 Department of Soil Science, Faculty of Agricultural Sciences and Technology, Bahauddin Zakariya University, Multan, Punjab Pakistan

**Keywords:** Diseases, Cancer

## Abstract

Recently, natural photosensitizers, such as berberine, curcumin, riboflavin, and emodin, have received more and more attention in photodynamic therapy. Tanshinone I (TanI) is extracted from a traditional Chinese herb Danshen, and exhibits many physiological functions including antitumor. TanI is a photoactive phytocompounds, but no work was tried to investigate its potential photodynamic effect. This study evaluated the cytotoxicity induced by the photodynamic effect of TanI. The photochemical reactions of TanI were firstly investigated by laser flash photolysis. Then breast cancer cell line MDA-MB-231 was chosen as a model and the photodynamic effect of TanI on cancer cell was evaluated by MTT assay and flow cytometry. The results showed that TanI could be photoexcited by its UV–Vis absorption light to produce ^3^TanI* which was quickly quenched by O_2_. MTT assay showed that the photodynamic effect of TanI resulted in more obvious inhibitive effect on cell survival and cell migration. Besides, the photodynamic effect of TanI could induce cell apoptosis and necrosis, lead to cell cycle arrest in G2, increase intracellular ROS, and decrease the cellular Δψm. It can be concluded that the photodynamic effect of TanI can obviously enhance the cytotoxicity of TanI on MDA-MB-231 cells in vitro, which indicated that TanI might serve as a natural photosensitizer.

## Introduction

Photodynamic therapy (PDT) has been widely used as a well-known therapeutic method to treat cancer, infections, and other diseases^1–3^. PDT involves a chemical process that a photosensitizer (PS) is excited by light to generate its triplet state which can react with O_2_ via energy transfer to generate singlet oxygen (^1^O_2_) or experience electron transfer to form reactive radicals^3^. PS plays a critical role in PDT. Recently, photoactive phytocompounds have received more and more attention in PDT because they are environmentally sustainable and lack major side effects^4^. Many phototoxic compounds were discovered in various plant families and had already been used as PS in PDT, such as berberine, curcumin, hypericin, riboflavin and emodin^5–7^.

Tanshinone I (TanI) is an extract from a traditional Chinese herb Danshen (*Salvia miltiorrhiza Bunge*) which has been successfully used for treating coronary heart diseases in clinics^8–11^. Many studies had also reported that TanI showed anti-tumor effects on various cancers, including ovarian cancer, glioblastoma, gastric cancer, hepatocellular carcinoma and breast cancer cells^12–17^. TanI has UV–visible absorption in the range of 200–600 nm and the maximum absorption in visible light spectrum is centered at blue light area. Previous study reported that TanI could be photo-excited by 532 nm laser pulse to generate its triplet state, which indicated that TanI is a photoactive molecule^18^.

Although TanI was proved to have antitumor effect on breast cancers via different pathways, no attempts were made to use TanI as a photosensitizer in breast cancer treatment. Therefore, this study attempted to investigate the photodynamic effect of TanI on the breast cancer MDA-MB-231 cells. In this study, the photoreactions of TanI under the excitation of 266 nm and 355 nm laser pulse were at first investigated by laser flash photolysis. And then the generation of ^1^O_2_ induced by TanI photosensitization was evaluated. At last, the photodynamic effect of TanI on MDA-MB-231 cells was investigated from cellular level by MTT, wound-healing assay and flow cytometry.

## Materials and methods

### Materials and reagents

All chemical reagents in this study were purchased and used directly without further purification. 3-(4,5-dimethylthiazol-2-yl)-2,5-diphenyltetrazolium bromide (MTT, ≥ 98.0%), TanI (97%), dimethyl sulfoxide (DMSO, ACS, ≥ 99.9%), and 1,3-diphenylisobenzofuran (DPBF, 98%) were purchased from Shanghai Aladdin Biochemical Technology Co., Ltd. Dulbecco’s modified Eagle’s medium (DMEM) without phenol red were purchased from Sangon Biotech (Shanghai) Co., Ltd. Fetal bovine serum (FBS) was purchased from Shanghai Zhongqiao Xinzhou Biotechnology Co., Ltd. Trypsin and all the KITs used in this study were purchased from Shanghai Biyuntian Biotechnology Co., Ltd.

Light lamp board (30 W, 14 cm × 20 cm) with wavelength centered at 460 nm (460 ± 10 nm) were purchased from Xuzhou Aijia Electronic Technology Co., Ltd (China). The distance between light source and 96-plates was set at 24 cm. The light radiation flux density is 10 ± 2 W/m^2^ and the luminous flux is 20 ± 5 lm. According to the light radiation flux density, 30 min light irradiation could bring light doses of 1.8 J/cm^2^.

### Cell culture

Breast cancer MDA-MB-231 cell line was purchased from the American Type Culture Collection (ATCC). The cells were cultured in DMEM without phenol red which was supplemented with 10% fetal bovine serum (FBS) and 100 units/mL penicillin in a humidified atmosphere of 5% CO_2_ at 37 °C.

### Laser flash photolysis

The laser flash photolysis setup has been previously described^25^. Laser flash photolysis experiments were carried out using Nd:YAG laser of 266 nm (30 mJ per pulse) and 355 nm (50 mJ per pulse) light pulses with a duration of 5 ns used as the pump light source. A xenon lamp was employed as analyzing light source. The laser and analyzing light beam passed perpendicularly through a quartz cell with an optical path length of 10 mm. The transmitted light entered a monochromator equipped with an R955 photomultiplier. The output signal from the Agilent 54830B digital oscilloscope was transferred to a personal computer for data treatment. The intensity of laser pulse was measured with a laser energy meter (Coherent EPM1000). TanI was dissolved in acetonitrile and the solutions were saturated with high-purity N_2_ (≥ 99.99%), N_2_O (≥ 99.5%), or O_2_ (≥ 99.5%) for different purposes by bubbling for at least 20 min prior to experiments.

### Detection of singlet oxygen in cell-free system

^1^O_2_ was detected by utilizing DPBF according to previous publications with modification^19,20^. In this study, DPBF (1 mM) and TanI (0 mM or 1 mM) were dissolved in DMSO and bubbled with different gases. LED light source (30 W) with wavelength centered at 520 nm was used. DPBF contents in different samples were firstly irradiated by LED light source for the designed time intervals, and then transferred to automatic injection bottles. Finally, the samples in automatic injection bottleswere analyzed by high performance liquid chromatography system (HPLC, Agilent 1260 Infinit II, Agilent Technologies, USA) with a C_18_ column (Agilent 5HC-C18(2), 4.6 × 250 mm, PN 588905-902, SN 555138, Agilent Technologies, USA). The mobile phase was a mixture of distilled water and acetonitrile (5:95, v/v). The loading amount was 0.1 mL. The flow rate of mobile phase was 0.8 mL/min and the detection wavelength was set as 410 nm.

### Uptake of TanI by MDA-MB-231

Cellular uptake of TanI was analyzed by flow cytometry. MDA-MB-231 cells were seeded into 6-well plates at a density of about 2 × 10^4^ per well. After being cultured for 24 h, the cells were treated with culture medium containing 10 μg/mL TanI (36 μM) and further incubated for different time intervals (1, 2, 3, 4, 6 and 8 h). The cells were washed with PBS for three times and then were detached with trypsinization. Subsequently, the cells were collected via centrifugation at 2000 rpm for 3 min. After being washed with PBS once, the cells were resuspended in 1 mL PBS and analyzed by flow cytometry (ACEA Novocyte 3110, USA). The washing and centrifugation processes were conducted in room temperature conditions and the PBS used herein was preheated at room temperature.

### Cytotoxicity assays

The effect of TanI photosensitization on MDA-MB-231 cells was investigated by MTT assay. In brief, MDA-MB-231 cells were seeded in 96-well plates with cell density of 8 × 10^3^ cells per well. After 24 h incubation, the cells were treated with culture medium containing different concentrations of TanI and further incubated for 4 h. Next, the cells were irradiated by light for 30 min. After further incubation for 24 h or 48 h, 50 μL MTT solution (1 mg/mL) was added into each well and the cells was incubated for 4 h. The supernatant was then carefully removed, and DMSO (150 μL) was added to each well. The plates were then placed in a shaking table and keeping slightly shaking for 30 min. At last, a microplate reader (Thermo Scientific, Multiskan GO, USA) was utilized to measure the OD values of each well at 540 nm.

### Evaluation of cell death mechanisms

The cell death mechanism was analyzed by flow cytometry with an Annexin V-FITC apoptosis detection kit (Beyotime, China). In the study, MDA-MB-231 cells were seeded into 6-well plates at a density of 2 × 10^4^ per well. After 24 h incubation, the cells were treated with 2 mL culture medium containing 1 μg/mL TanI (3.6 μM). After being further incubated for 4 h, the cells were treated by 460 nm light for 30 min, and further incubated for 24 h. The supernatant was then carefully removed and the cells were washed with PBS twice. After being detached with trypsinization, the cells were washed with PBS and collected by centrifugation, and subsequently double stained with Annexin V and PI (propidium iodide). Finally, the prepared cells were measured with flow cytometry by analyzing 20 000 cells.

### Intracellular ROS assay

The change of intracellular ROS was investigated by flow cytometry with a ROS Assay Kit (Beyotime, China). Typically, MDA-MB-231 cells at a density of 2 × 10^4^ cells per well were firstly seeded into 6-well culture plates and then cultured for 24 h. Next, 2 mL medium containing 1 μg/mL TanI (3.6 μM) was added to each well. After being further incubated for 4 h, the cells were irradiated by 460 nm light for 30 min. Immediately, the cells were detached with trypsinization, washed with PBS and collected by centrifugation, successively resuspended in serum‐free medium containing 10 μmol/L DCF-DA and incubated for 20 min in the dark with rocking every 5 min. Finally, the prepared cells were measured with flow cytometry by detecting the fluorescence intensity of DCF.

### Detection of mitochondria membrane potential

The change of cellular mitochondria membrane potential was investigated by flow cytometry with a mitochondrial membrane potential assay kit with JC-1 (Beyotime, China). Basically, MDA-MB-231 cells were seeded in a 6-well plate at 2 × 10^4^ cells per well and incubated for 24 h. The media was replaced by 2 mL fresh ones containing 1 μg/mL TanI (3.6 μM). After being further incubated for 4 h, the cells were irradiated by 460 nm LED light for 30 min. After irradiation, the cells were incubated for another 24 h and were then detached with trypsinization. The cells were then collected by centrifugation and stained using JC-1 according to the manufacturer’s protocol. Finally, the prepared cells were measured with flow cytometry by analyzing 20 000 cells.

### Cell cycle distribution

The change of cell cycle distribution was investigated by flow cytometry with a Cell Cycle and Apoptosis Analysis Kit (Beyotime, China). Briefly, MDA-MB-231 cells were seeded into 6-well plates at a density of 2 × 10^4^ per well and incubated for 24 h. The media was then replaced by 2 mL fresh ones containing 1 μg/mL TanI (3.6 μM). After being further incubated for 4 h, the cells were irradiated by 460 nm LED light for 30 min. After irradiation, the cells were incubated for another 24 h and were then detached with trypsinization. After that, the cells were collected by centrifugation and washed with PBS once. At last, the cells were treated according to the manufacturer’s protocol. The prepared cells were analyzed via flow cytometry.

## Results

### Characterization of the triplet state of TanI

**Figure 1:**
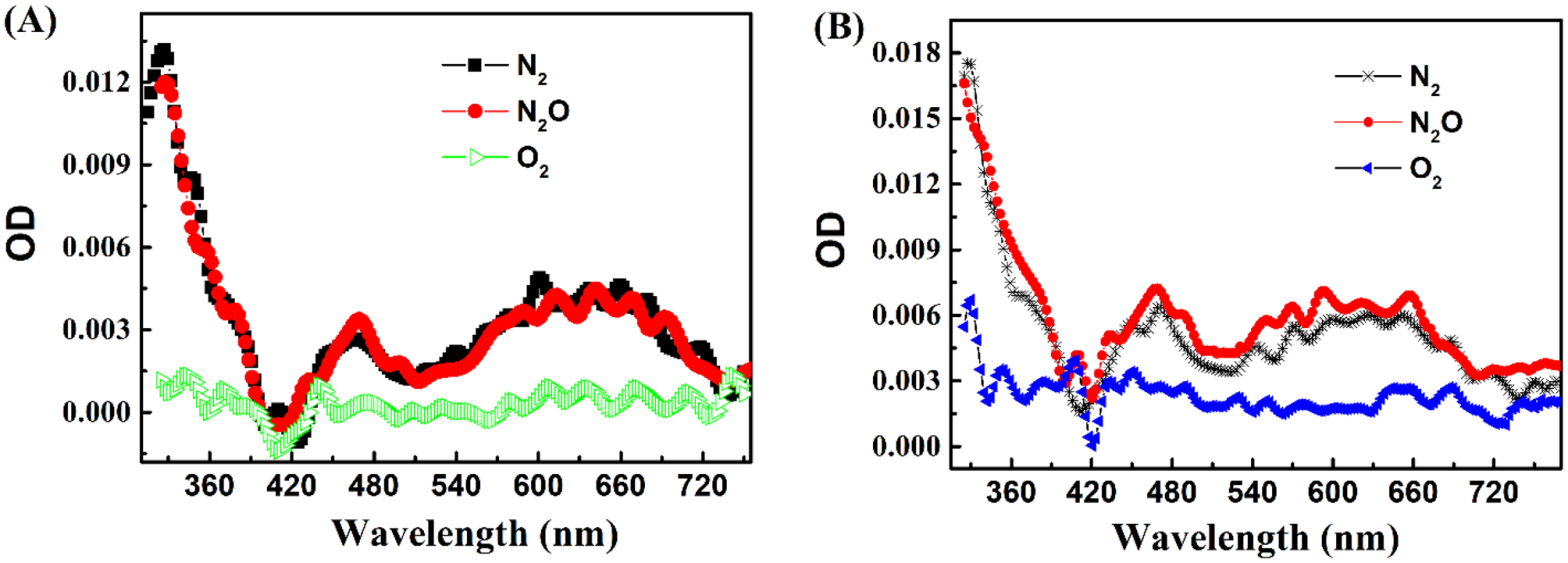
Transient absorption spectra recorded at 0.1 μs after the laser pulse in the 266 nm (**A**) and 355 nm (**B**) laser flash photolysis of acetonitrile solution containing 0.05 mM TanI saturated with N_2_, O_2_ and N_2_O, respectively.

At first, laser flash photolysis of TanI with 266 nm and 355 nm laser pulses was explored in order to understand the photoreactions of TanI under the irradiation of its absorption light at different wavelengths. As shown in Fig. 1, photo-irradiation of N_2_-saturated TanI acetonitrile solution with 266 nm and 355 nm laser pulses could generate similar transient absorption spectra but with different intensity. The transient absorption spectra had three characteristic absorption peaks which were centered at 330 nm, 470 nm and 630 nm, respectively. Both transient absorption spectra could not be influenced by solvated electron scavenger N_2_O, but could be efficiently eliminated by triplet state quencher O_2_. Meanwhile, the kinetic decay curves at 330 nm, 470 nm and 630 nm were obviously accelerated by O_2_, but kept nearly unchanged under the saturation of N_2_O (sFig. 1). These results suggested that the transient absorption spectra obtained in N_2_-saturated acetonitrile solution should be assigned to the absorption of ^3^TanI* and TanI experienced photoexcitation to produce its triplet states (^3^TanI*) by 266 nm or 355 nm laser pulses.

### Generation of singlet oxygen in noncellular system

**Figure 2:**
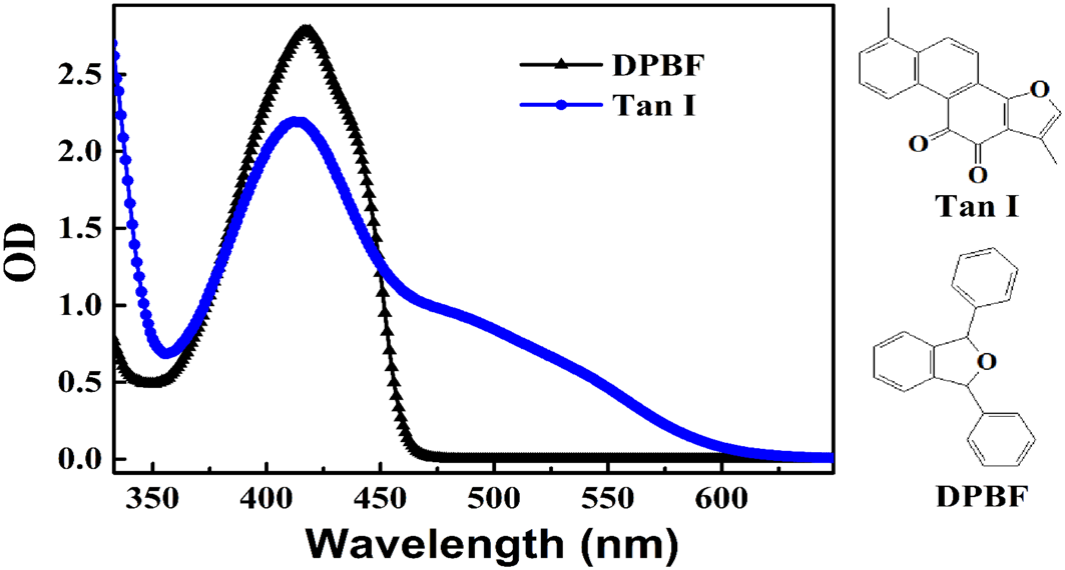
The UV–Vis absorption spectra of TanI and DPBF in DMSO.

The triplet state of a photosensitizer can be easily quenched by O_2_ to generate ^1^O_2_ which was proved to play a critical role in PDT^28,29^. DPBF was a widely-used ^1^O_2_ probe and could react irreversibly with O_2_ to cause the photodegradation of DPBF. ^1^O_2_ was usually detected by analyzing the change of DPBF via measuring its absorption at 410 nm^26,27^. In this study, the absorption of DPBF at 410 nm was seriously overlapped by that of TanI (Fig. 2). Therefore, HPLC was utilized to analyze the change of DPBF. As shown in Fig. 2, DPBF could not absorb light with wavelength beyond 470 nm. Therefore, a LED light source with wavelength centered at 520 nm was chosen in order that the light could lead to the photo-excitation of TanI, and meanwhile could not result in the direct photodegradation of DPBF.

**Figure 3:**
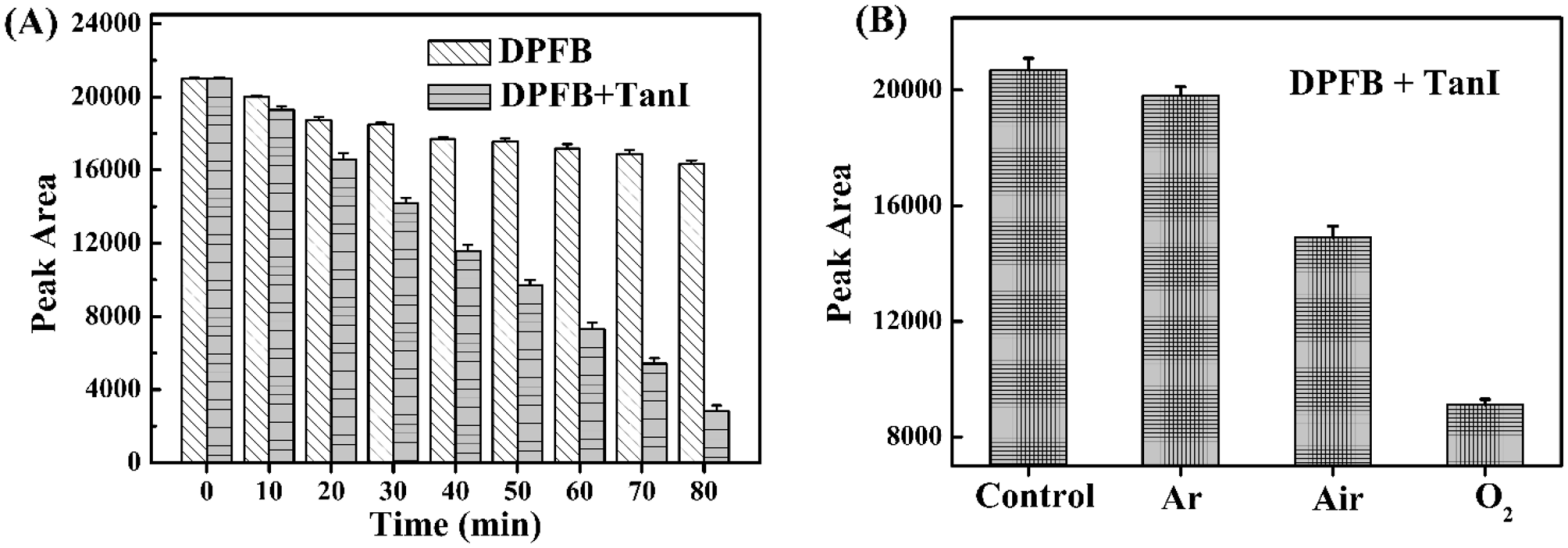
(**A**) The content of DPBF in the solutions containing DPBF (1 mM) and TanI (0 and 1 mM) under the irradiation of LED light with wavelength centered at 520 nm for different time, measured by HPLC immediately after light irradiation. (**B**) The content of DPBF in the solutions containing DPBF (1 mM) and TanI (1 mM) without (control) or with the irradiation of LED light with wavelength centered at 520 nm for 30 min, measured by HPLC immediately after light irradiation. The light-treated solutions were bubbled with Ar, air and O_2_, respectively.

As shown in Fig. 3A, DPBF content still decreased without TanI when the irradiation time increased, which suggested that the light source chosen herein might emit a part of light that could be absorbed by DPBF, and induced the photodegradation of DPBF in some extent. In comparison, the presence of TanI could remarkably accelerate the decrease of DPBF when the other conditions were kept unchanged.

The effect of O_2_ on the photodegradation of DPBF was then investigated. The samples with different O_2_ content were obtained by being saturated with Ar, air and O_2_, respectively. The result showed that the photodegradation of DPBF in the presence of TanI was an O_2_-dependent way (Fig. 3B). It was explainable that the increase of O_2_ content could enhance ^1^O_2_ generation via energy transfer between ^3^TanI* and O_2_, thereby accelerating the photodegradation of DPBF. In summary, the photodynamic effect of TanI could generate ^1^O_2_ in an irradiation time-dependent and O_2_-dependent way.

### Cellular uptake of TanI by MDA-MB-231 cells

**Figure 4:**
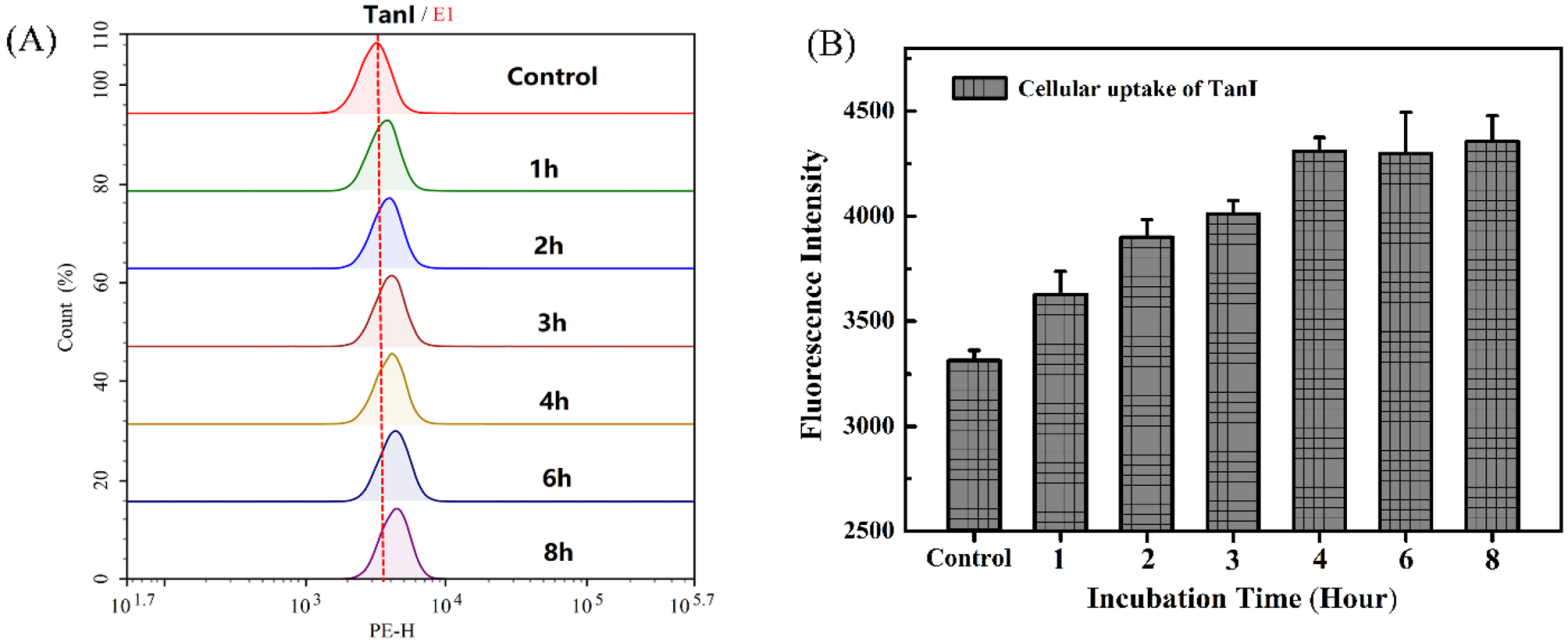
The uptake of TanI by MDA-MB-231 cells incubated with 10 μg/mL TanI (36 μM) for different time (1 h, 2 h, 3 h, 4 h, 6 h and 8 h) studied by flow cytometry.

The cellular uptake of TanI by MDA-MB-231 cells was explored by analyzing the fluorescence of TanI via flow cytometry. The results showed that the cellular uptake of TanI increased as the incubation time increased from 1 to 8 h (Fig. 4). When the incubation time exceeded 4 h, the increase of cellular uptake was not obvious. The result suggested that TanI could efficiently enter into intracellular space and the cellular uptake might be close to saturated state when the incubation time exceeded 4 h.

### The change of cell growth status

**Figure 5:**
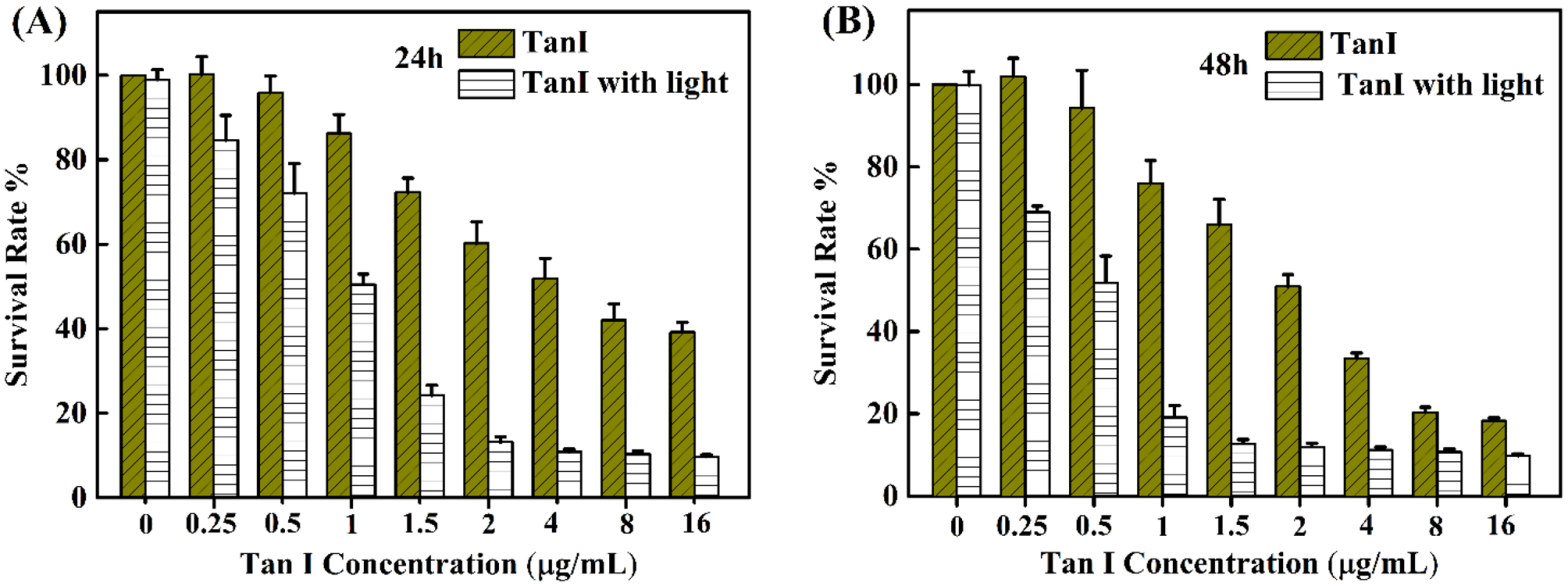
The survival rate of MDA-MB-231 cells incubated with different concentrations of TanI for 4 h and followed by 460 nm light irradiation for 30 min, measured at 24 h and 48 h, respectively (All samples were run in triplicate).

MTT assays were conducted to investigate the photodynamic effect of TanI on the survival of MDA-MB-231 cells. As shown in Fig. 5, the survival rate of MDA-MB-231 cells could not be affected by light irradiation without TanI. It was reported in many studies that TanI itself could exert antitumor effect^19,20^. Therefore, the inhibition of MDA-MB-231 cells survival in a dose-dependent way was also observed in this study. The IC50 of TanI at 24 h and 48 h were 5.7 µg/mL and 2.5 µg/mL, respectively. In contrast, light irradiation obviously enhanced the inhibition effect of TanI on the cell’s survival, leading to the IC50 decreased to 0.857 µg/mL and 0.456 µg/mL at 24 h and 48 h respectively. The results suggested that the photodynamic effect of TanI efficiently enhanced the cytotoxic effect of TanI on MDA-MB-231 cells.

The light source used in this study could cause TanI photodecomposition (sFig. 2A). In order to exclude the effect of photodecomposition products, the culture mediums containing TanI were irradiated by light before they were added into 96 wells-plates. In this way, the cells were separated from the photodynamic effect of TanI. The 48 h MTT assay showed that light irradiation slightly reduced the cytotoxic effect of TanI on MDA-MB-231 cells (sFig. 2B). Therefore, it could be inferred that it was the photodynamic effect of TanI rather than photodecomposition products that enhanced the cytotoxic effect of TanI on MDA-MB-231 cells.

Moreover, the photodynamic effect of TanI on cell migration was evaluated by wound-healing assay. As shown in sFig. 3, MDA-MB-231 cells in separate light irradiation group showed similar scratch wound-healing ability with the cells in control group. The addition of TanI slightly inhibited cell migration as compared with the TanI-free groups. However, the combination of TanI and light irradiation distinctly inhibited the recovery of scratch interval, which suggested that the photodynamic effect of TanI could inhibit the migration of MDA-MB-231 cells in vitro.

### The change of cell apoptosis and necrosis

**Figure 6:**
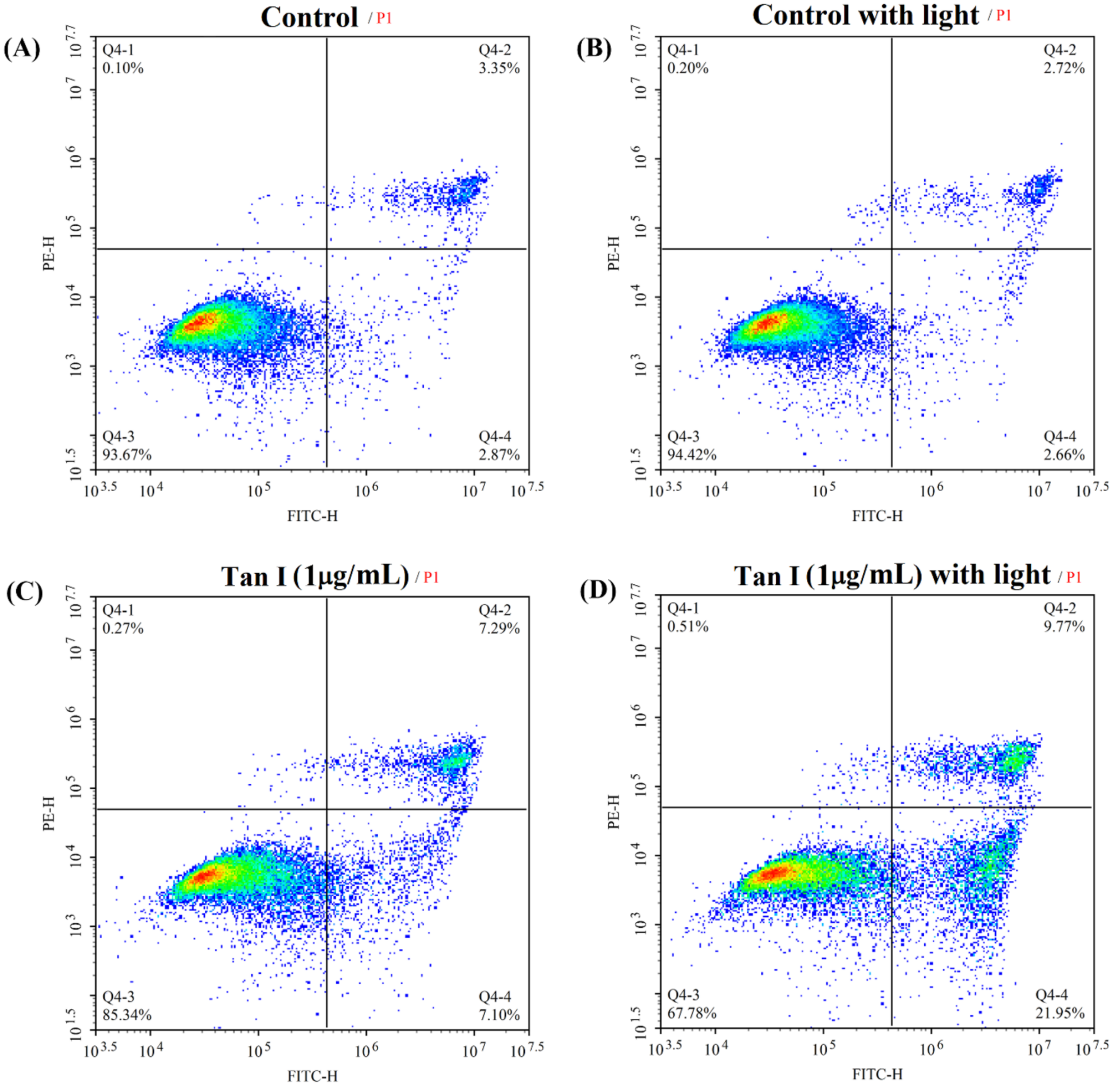
The apoptosis and necrosis of MDA-MB-231 cells incubated with 1 μg/mL TanI (3.6 μM) for 4 h and followed by 460 nm light irradiation for 30 min, measured by flow cytometry after further incubation for 24 h.

The photodynamic effect of TanI on cell apoptosis and necrosis was investigated by flow cytometry. The result showed that light irradiation had little effect on cell apoptosis and necrosis as compared with control group (Fig. 6A,B). The percentage of apoptotic and necrotic cells was slightly increased by TanI from 6 to 14% as compared with control group (Fig. 6C), which was assigned to the antitumor effect of TanI itself. However, TanI-treatment followed by light irradiation obviously increased the percentage of apoptotic and necrotic cells from 14 to 31% as compare with TanI-treatment group (Fig. 6C,D). It could be inferred that the photodynamic effect of TanI led to cell death via efficiently inducing cell apoptosis and necrosis.

### The change of intracellular reactive oxygen species

**Figure 7:**
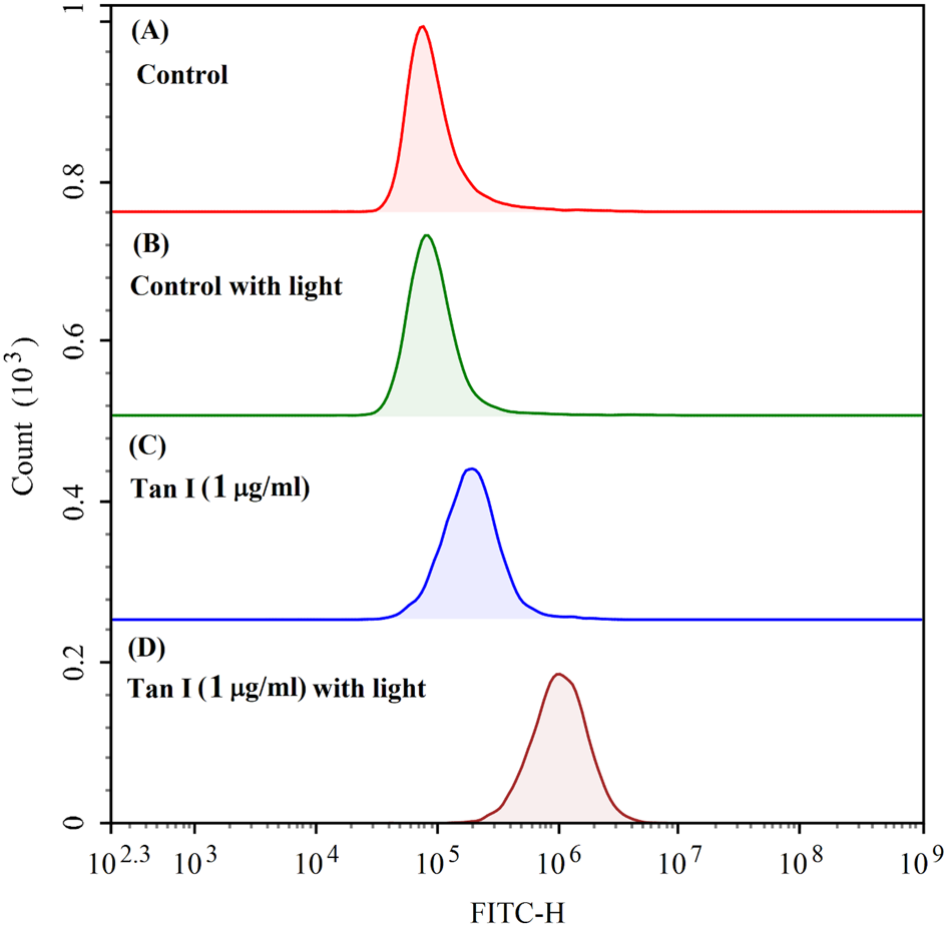
The intracellular ROS level in MDA-MB-231 cells incubated with 1 μg/mL TanI (3.6 μM) for 4 h and followed by 460 nm light irradiation for 30 min, measured by flow cytometry immediately after light irradiation. (**A**) control group; (**B**) light irradiation; (**C**) TanI-trearment; (**D**) TanI-treatment followed by light irradiation.

The photodynamic effect of TanI on intracellular ROS level in MDA-MB-231 cells was investigated by utilizing ROS probe DCFH-DA. The result showed that ROS level in MDA-MB-231 cells was not changed by light irradiation itself (Fig. 7A,B). As compared with control group, TanI itself could also increase the cellular ROS. It was reported that TanI could inhibit breast cancer cells via many different mechanisms, including via causing intracellular ROS accumulation^19,20^. Therefore, it was explainable that TanI-treatment herein could cause the elevation of ROS level in MDA-MB-231 cells. However, TanI-treatment followed by light irradiation could further increase the DCF fluorescence intensity in MDA-MB-231 cells as compared with separate TanI-treatment group (Fig. 7C,D). It could be inferred that the photodynamic effect of TanI could further increase the intracellular ROS accumulation in MDA-MB-231 cells.

### The change of mitochondrial membrane potential

**Figure 8:**
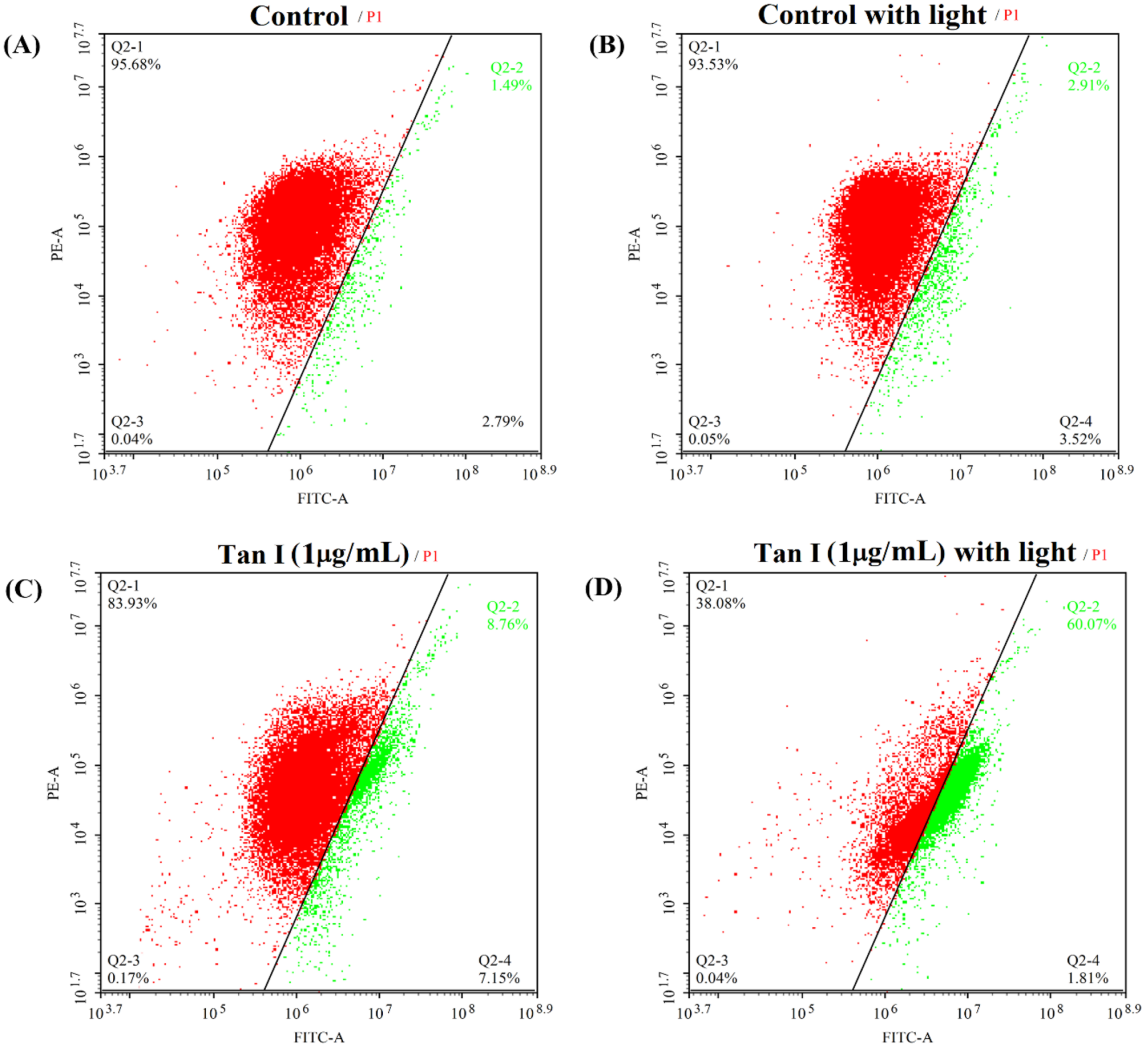
The mitochondrial membrane potential of MDA-MB-231 cells incubated with 1 μg/mL TanI (3.6 μM) for 4 h and followed by 460 nm light irradiation for 0 or 30 min, measured by flow cytometry after further incubation for 24 h.

The decrease of mitochondrial membrane potential (ΔΨ_m_) is a landmark event in the early stage of apoptosis^30^. The change of ΔΨ_m_ for MDA-MB-231 cells was detected by flow cytometry with a fluorescent probe JC-1. It was found that the cellular ΔΨ_m_ in MDA-MB-231 cells could be slightly reduced by the separate action of light irradiation or TanI, respectively (Fig. 8). However, as compared with TanI-treatment group, the ΔΨ_m_ of MDA-MB-231 cells was remarkably reduced by the synergistic action of TanI and light irradiation. It could be inferred that the photodynamic effect of TanI could induce the decrease of ΔΨ_m_ in MDA-MB-231 cells.

### The change of cell cycle distribution

**Figure 9:**
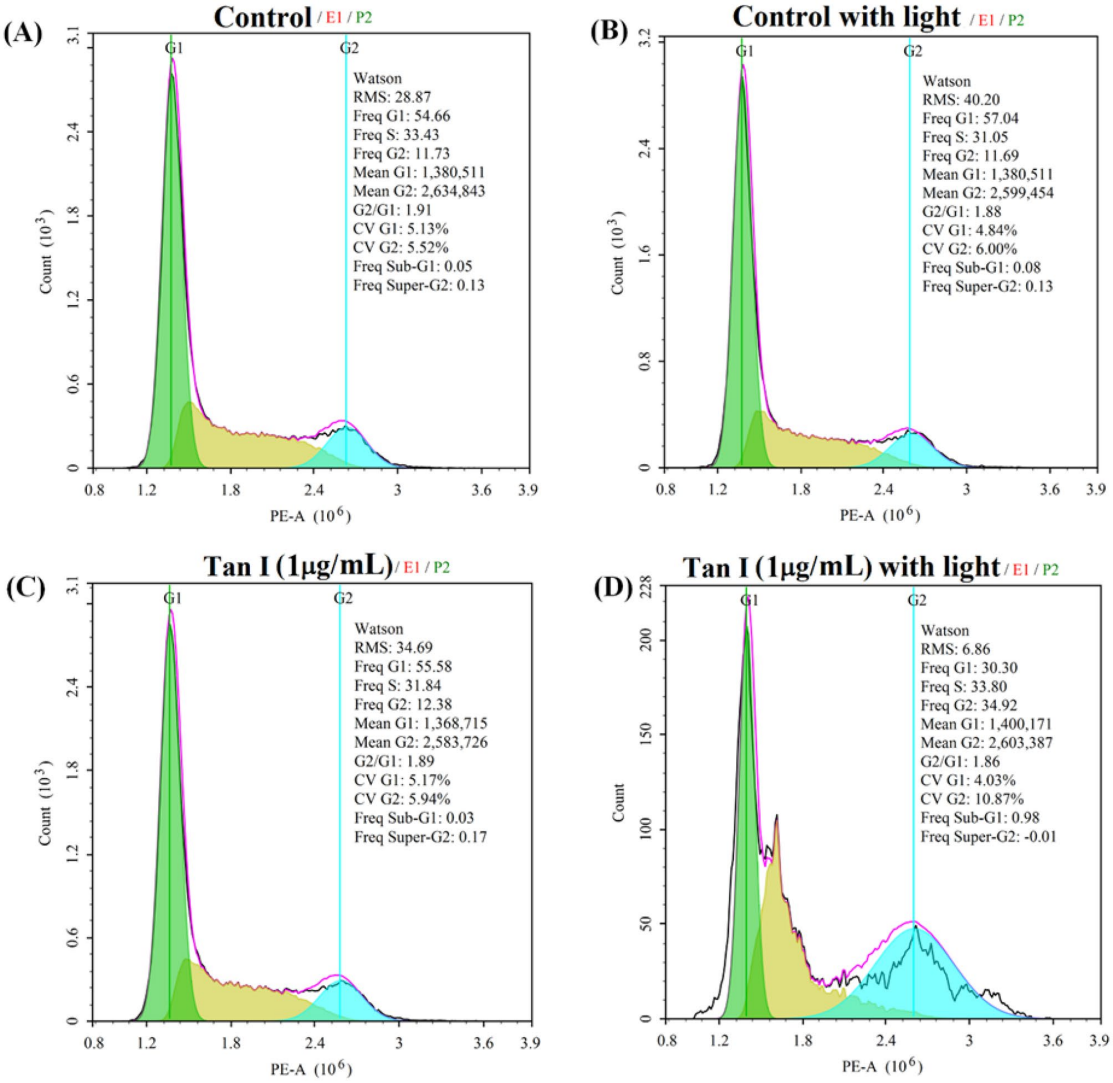
The cell cycle distribution of MDA-MB-231 cells incubated with 1 μg/mL TanI (3.6 μM) for 4 h and followed by 460 nm light irradiation for 30 min, measured by flow cytometry after further incubation for 24 h.

The photodynamic effect of TanI on cell cycle distribution was investigated by flow cytometry. As shown in Fig. 9, the cell cycle distribution of MDA-MB-231 cells remained nearly unchanged when light irradiation or TanI was conducted separately as compared with control group. However, as compared with the other groups, the synergistic action of light irradiation and TanI could significantly reduce the proportion of cells in S and G1 phase and increase that in G2 phase. The result suggested that the photodynamic effect of TanI could result in the arrest of MDA-MB-231 cells in G2 phase.

## Discussion

Many photosensitive molecules from natural resources, such as curcumin, berberine, hypericin and riboflavin, were proved to have photodynamic effect and were explored as photosensitizers in PDT^5–7^. These molecules usually come from edible plants, medical herbs, or clinical drugs, and are proved to be low toxic^4,21^. TanI, belonging to this kind of molecules, is not only a photosensitive molecule, but also one of the bioactive components of Danshen which is widely used in traditional Chinese medicine.

Laser flash photolysis of TanI by 266, 355 and 532 nm laser pulses could all lead to the generation of ^3^TanI*, which indicated that TanI could be photoexcited by its UV–Vis absorption light at different wavelengths. The triplet state of a photosensitizer, which is a prerequisite for PDT, can form reactive radicals via electron transfer (Type I reactions) or react with O_2_ via energy transfer to generate ^1^O_2_ (Type II reactions). Most evidences supported that Type II reactions was a prevalent molecular proesses in PDT^22,23^. In this study, it was found that the generated ^3^TanI* could be efficiently quenched by O_2_ via energy transfer to generate ^1^O_2_ (Type II reactions) in an irradiation time-dependent and O_2_-dependent way. ^1^O_2_ was thought to be the most destructive ROS to living organisms and was proved by many evidences to play a key role in the molecular processes initiated by PDT^22,23^. Therefore, the photodynamic effect of TanI on breast cancers via Type II reactions was anticipated. And our results demonstrated that the photodynamic effect of TanI could efficiently inhibit the survival and migration of MDA-MB-231 cells, and cause cell death via efficiently inducing cell apoptosis and necrosis. Generally speaking, inducing the increase of intracellular ROS level is the main consequence of photodynamic therapy^2,3^. Although TanI itself could induce the intracellular ROS accumulation, the photodynamic effect of TanI could further increase the intracellular ROS level in MDA-MB-231 cells.

The destruction of mitochondrial integrity is the initial step in the intrinsic pathway of apoptosis induction and usually characterized by the decrease of ΔΨ_m_^24^. In this study, the photodynamic effect of TanI magnificently decreased the ΔΨ_m_ in MDA-MB-231 cells. It is known that mitochondria are one of many critical sites which are vulnerable to the attack of ROS^25–28^. It was possibly that the accumulated ROS induced by the photodynamic effect of TanI might disrupt mitochondrial integrity and lead to the decrease of ΔΨ_m_, eventually trigger the signal of cellular apoptosis. G2/M phase has a G2/M DNA damage checkpoint which is responsible for preventing the cell from entering mitosis (M-phase) when DNA is damaged, providing an opportunity for repair and stopping the proliferation of damaged cells^29,30^. In this work, the photodynamic effect of TanI led to the distinct cell arrest in G2 phase, which suggested that the photodynamic effect of TanI might pose damage to DNA, thereby leading to the arrest of cells in G2/M phase.

Many natural photosensitizers, such as riboflavin, berberine, curcumin, had been reported to have photodynamic effect on tumor or bacteria^5,7,31,32^. These natural photosensitizers similar to TanI usually have strong absorption in blue light zone, and blue light sources were usually used in some of these studies. Blue light had been used in many studies for some sub-epidermal diseases, such as psoriasis, acne, actinic keratosis, cutaneous infections^33–36^. However, blue light indeed has weak tissue penetration ability, which limits the application of these natural photosensitizers in PDT. Fortunately, upconversion luminescent materials which can absorb infrared light and emit visible light (including blue light), had been successfully used to overcome the defect of blue light^37–40^, which may provide an opportunity to extend the application TanI in PDT. Anyhow, this study may provide a theoretical reference for TanI as a natural photosensitizer in PDT to treat tumor, infection, or some skin diseases.

## Conclusions

In this study, it was found that TanI could be photoexcited to produce ^3^TanI* by its UV–Vis absorption light at different wavelengths and the quench of ^3^TanI* by O_2_ could lead to the generation of ^1^O_2_. The growth and migration of MDA-MB-231 cells could be obviously inhibited by the photodynamic effect of TanI. Moreover, the photodynamic effect of TanI could cause cell death via inducing cell apoptosis and necrosis. Besides, it was also proved that the photodynamic effect of TanI could increase the intracellular ROS, reduce ΔΨ_m_ and induce the arrest of MDA-MB-231 cells in G2 phase. This study confirms that the photodynamic effect of TanI can efficiently enhance the cytotoxic effect of TanI on MDA-MB-231 cells, which may provide a proof-of-principle demonstration for the application of TanI in photodynamic therapy.

### Supplementary Information

Supplementary Figures.

## Data Availability

All data generated or analysed during this study are included in this published article and supplementary file.
