# Supplementary material for: The enhanced cytotoxicity on breast cancer cells by Tanshinone I-induced photodynamic effect
Source: Sci Rep. 2023 Oct 23;13:18107. doi: 10.1038/s41598-023-43456-5 (PMC10593796; doi:10.1038/s41598-023-43456-5)
Supplement: Supplementary file 1 — Supplementary Figures. [file 41598_2023_43456_MOESM1_ESM.docx]

**Supplymentry File**

Potentiation of Photodynamic Effects Induced by Tanshinone I on Breast Cancer Cells for Enhanced Cytotoxicity

Chen Fengchao^1^, Zhang Siya^1^, Yan Tongtong^1^, Wang Hongquan^2^, Li Jie^2^, Wang Qiang^2^, Li Kun^2^, Subhan Danish^3^*


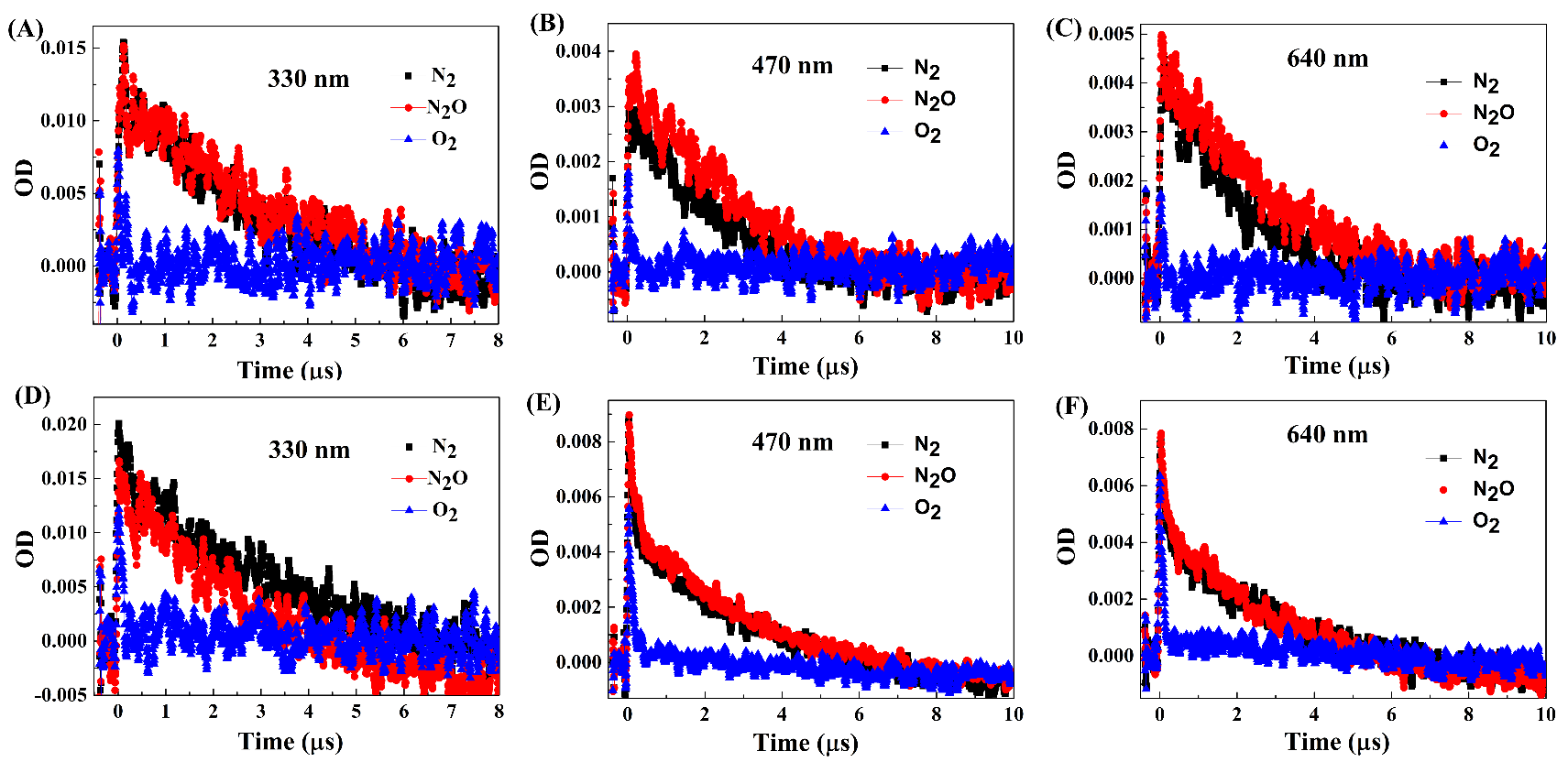


**sFigure 1.** The kinetic decay curves for ^3^Tan I* at different characteristic peaks were presented in the following figure. The kinetic decay curves at (A) 320 nm, (B) 470 nm and (C) 630 nm obtained from 266 nm laser flash photolysis of 0.05 mM TanI in acetonitrile saturated with N_2_, N_2_O, and O_2_, respectively. And the kinetic decay curves at (D) 320 nm, (E) 470 nm and (F) 630 nm obtained from 355 nm laser flash photolysis of 0.05 mM TanI in acetonitrile saturated with N_2_, N_2_O, and O_2_, respectively.

1. The kinetic decay curves for 3Tan I* at different characteristic peaks

**2. The effect of TanI photodecomposition on cell survival**


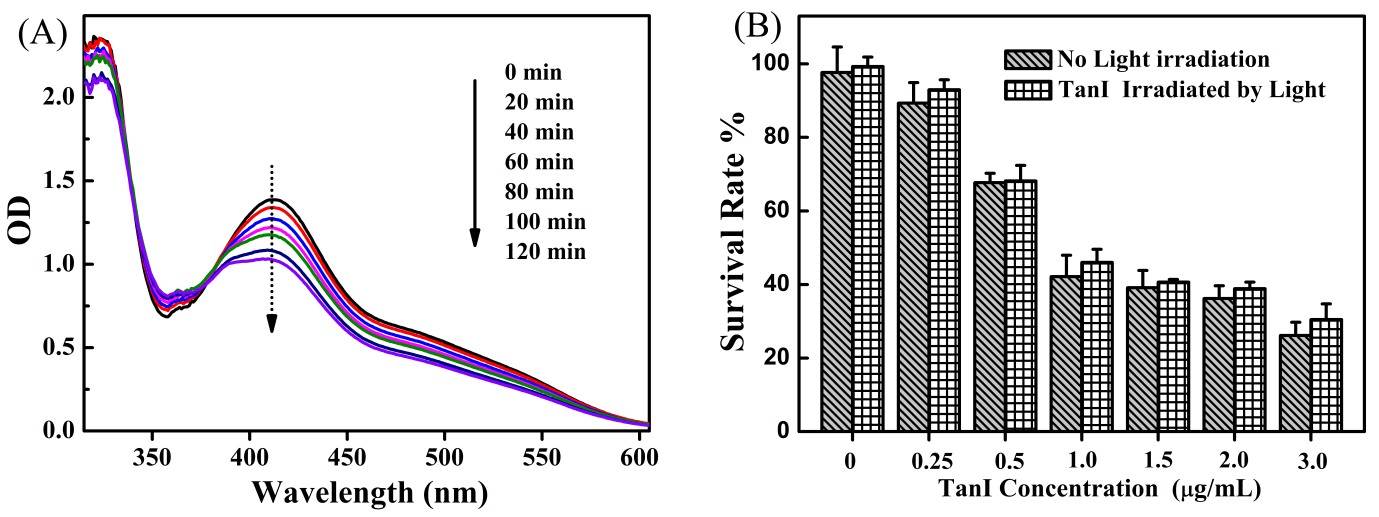


**sFigure.2** (A) the changes of UV-vis absorption spectra of Tan I (0.5 mg/mL) in DMSO which were recorded after 460 nm light irradiation for 0, 20, 40, 60, 80, 100, and 120 min, respectively. (B) The survival rate of MDA-MB-231 cells incubated with culture mediums containing different concentrations of Tan I which were irradiated by 460 nm light for 30 min before they were exposed to MDA-MB-231 cells, measured by MTT assay at 48 h (All samples were run in triplicate)

**3. The change of cell migration evaluated by wound-healing assay**


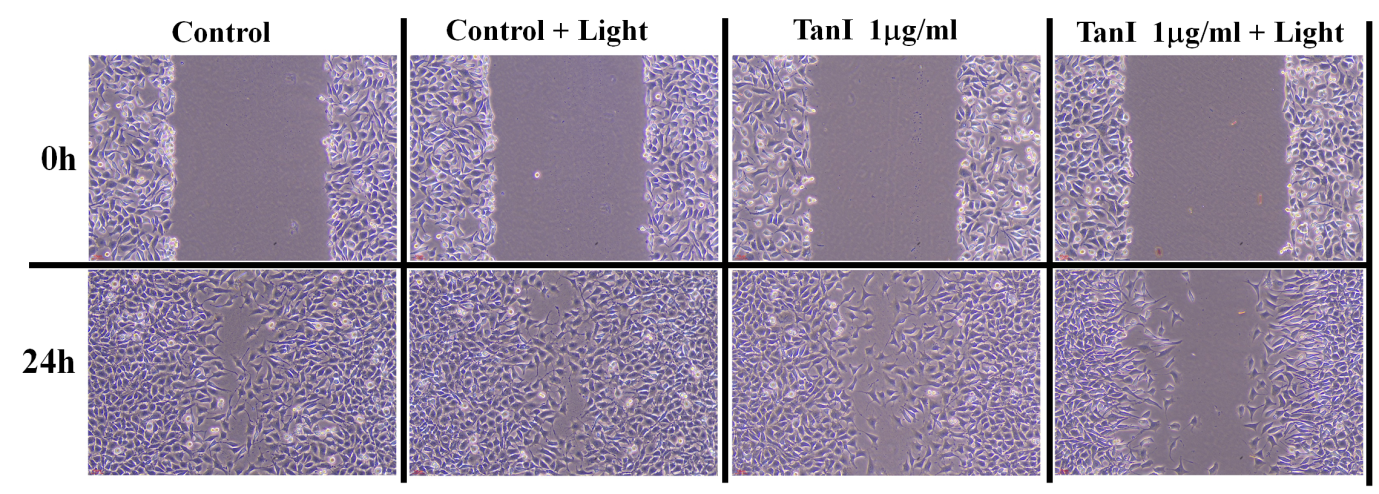


**sFigure 3.** The migration of MDA-MB-231 cells analyzed by wound-healing assay. The cell monolayer in 6-wells plate were scratched and then incubated with 1 μg/mL Tan I for 4 hours and followed by light irradiation for 30 min. Images of cell migration were captured by an inverted microscope at 0 and 24 h after scratching, respectively. MDA-MB-231 cells were seeded into 6-well plates at a density of 2 × 10^4^ per well and cultured in the incubator. After being cultured for 24 h, the cells formed a confluent monolayer. A sterile 200 μL needle was used to scratch at the bottom of 6-well plates to form a straight cell-free zone, after which the floating cells were washed off with PBS. Subsequently, the cells were treated with TanI (2.0 mL/well, 1μg/mL) and further incubated for 4 h. The cells were irradiated with 460 nm light for 30 min and further cultured in the incubator. The wound healing process was monitored through an inverted microscope. Images of cell migration were captured at 0 and 24 h, respectively.


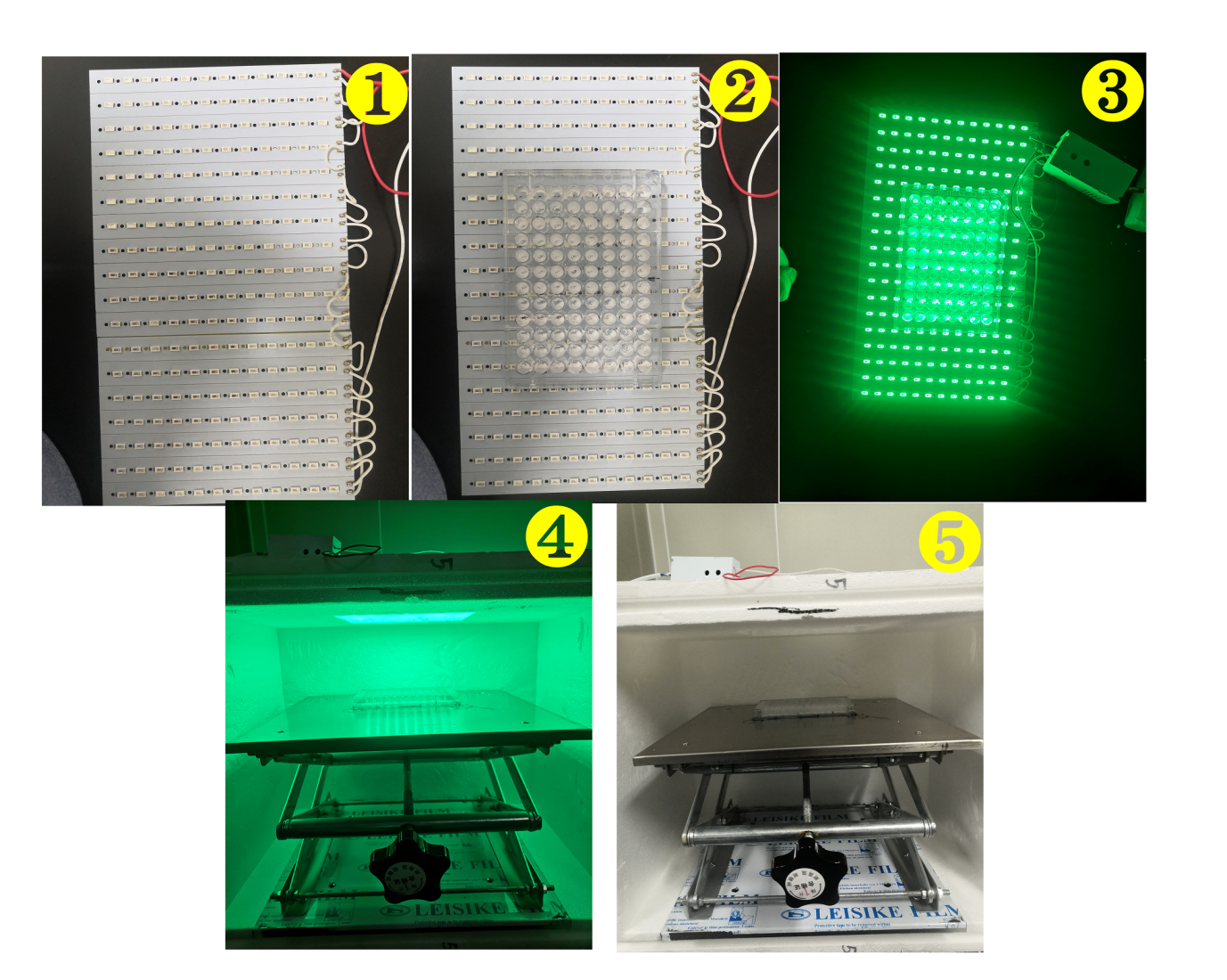
**4. The picture of the illumination setup**

**sFigure 4.** Light lamp board was chosen in this study was customized from the Xuzhou Aijia Electronic Technology Co., Ltd (China).Fig4.1, 4.2 and 4.3 are the pictures of light lamp boards with or without power; and Fig4.4 and 4.5 are the self-built integral irradiation device with or without power.
